# Noncoding RNAs in the Glycolysis of Ovarian Cancer

**DOI:** 10.3389/fphar.2022.855488

**Published:** 2022-03-30

**Authors:** Chunmei Zhang, Ning Liu

**Affiliations:** Department of Obstetrics and Gynecology, Shengjing Hospital of China Medical University, Shenyang, China

**Keywords:** circular RNAs, long non-coding RNAs, microRNAs, glycolysis, ovarian cancer

## Abstract

Energy metabolism reprogramming is the characteristic feature of tumors. The tumorigenesis, metastasis, and drug resistance of ovarian cancer (OC) is dependent on energy metabolism. Even under adequate oxygen conditions, OC cells tend to convert glucose to lactate, and glycolysis can rapidly produce ATP to meet their metabolic energy needs. Non-coding RNAs (ncRNAs) interact directly with DNA, RNA, and proteins to function as an essential regulatory in gene expression and tumor pathology. Studies have shown that ncRNAs regulate the process of glycolysis by interacting with the predominant glycolysis enzyme and cellular signaling pathway, participating in tumorigenesis and progression. This review summarizes the mechanism of ncRNAs regulation in glycolysis in OC and investigates potential therapeutic targets.

## 1 Introduction

Ovarian cancer (OC) is currently the most deadly gynecologic malignancy with insidious and rapidly progressive onset. Most patients have advanced pelvic and abdominal metastases by the time of diagnosis, and the 5-years survival rate is only 20–30% worldwide (Vafadar et al., 2020; DiSilvestro et al., 2021; Vergote et al., 2021). OC account for 5% of all cancer deaths in women (Yang et al., 2021; Youssef et al., 2021) due to the low survival rates resulting from late diagnosis. The standard treatment for OC is tumor resection combined with platinum-based chemotherapy. However, the majority with advanced disease will replase or even develop drug resistance, leading to curative failure and ultimately mortality (Giudice et al., 2021; Xie H et al., 2021; Xu et al., 2021). Therefore, it is essential to investigate new treatment options to improve the outcome of OC.

Tumorigenesis is considered an energy metabolic disease. Compared with metabolism of healthy and neoplastic cells, researchers found the oxidative phosphorylation pathway is dominant to provide ATP in normal cells, while the glycolytic pathway is the primary energy supply in tumor cells (Nakagawa et al., 2020; Tyagi et al., 2021). Even in the presence of sufficient oxygen, the glycolytic pathway, an alteration known as the Warburg effect, or aerobic glycolysis, accounts for over 95% of energy supply (Sun et al., 2018; Harris and Fenton 2019; Lu 2019). The altered glycolytic pathway is a characteristic difference between neoplastic and healthy cells (Icard et al., 2018). Tumor cells can produce more nucleotides, fatty acids, proteins, and ATP through enhanced aerobic glycolysis as the material basis for rapid proliferation and invasiveness (Poff et al., 2019). Meanwhile, the Warburg effect reduces reactive oxygen species production, improves cellular antioxidant capacity, and reduces apoptosis (Yue et al., 2016; Shulman and Rothman 2017; Yue et al., 2019). In addition, aerobic glycolysis can produce large amounts of lactic acid, which creates an acidic microenvironment to facilitate invasion and metastasis of the tumor cells (Schwartz et al., 2017; Tekade and Sun 2017; Chen et al., 2018).

Noncoding RNAs (ncRNAs) primarily include microRNAs (miRNAs), long noncoding RNAs (lncRNAs), and circular RNAs (circRNAs) (Jusic et al., 2020; Deogharia and Gurha 2021; Rahimian et al., 2021). The ncRNAs bind to multiple molecular targets to form regulatory networks in various biological activities, including initiating specific cellular biological responses, regulating gene expression, intracellular signaling, and epigenetic modifications (Ding et al., 2021; Ducoli and Detmar 2021). NcRNAs are involved in a variety of life activities such as regulation of gene expression, intracellular signaling and epigenetic modifications. Apart from participation in tumorigenesis, ncRNAs also account paramount role in the glycolytic process of tumors (Li Q et al., 2021; Lu et al., 2021; Park et al., 2021; Razavi et al., 2021; Wang et al., 2021). This review summarizes the possible molecular mechanisms of ncRNAs in the process of glycolysis and potentially effective targeted therapies for OC.

## 2 Glucose Metabolism in Neoplastic Cells

Reprogramming of energy metabolism is the hallmark of cancer. Healthy cells generally undergo glycolysis to produce lactate only under anaerobic conditions with limited energy production, while the glycolysis of tumor cells in aerobic conditions (Chandel 2021; Reinfeld et al., 2021). Although glycolysis produces low levels of ATP compared to oxidative phosphorylation, cancer cells can rapidly uptake the available ATP and intermediates from glycolysis for the transduction of the biosynthetic pathway (Bacigalupa and Rathmell 2020; Cao et al., 2020). The reprogrammed metabolism contributes to tumor cell metastasis, preventing apoptosis and promoting other malignant features.

### 2.1 Warburg Effect

**FIGURE 1:**
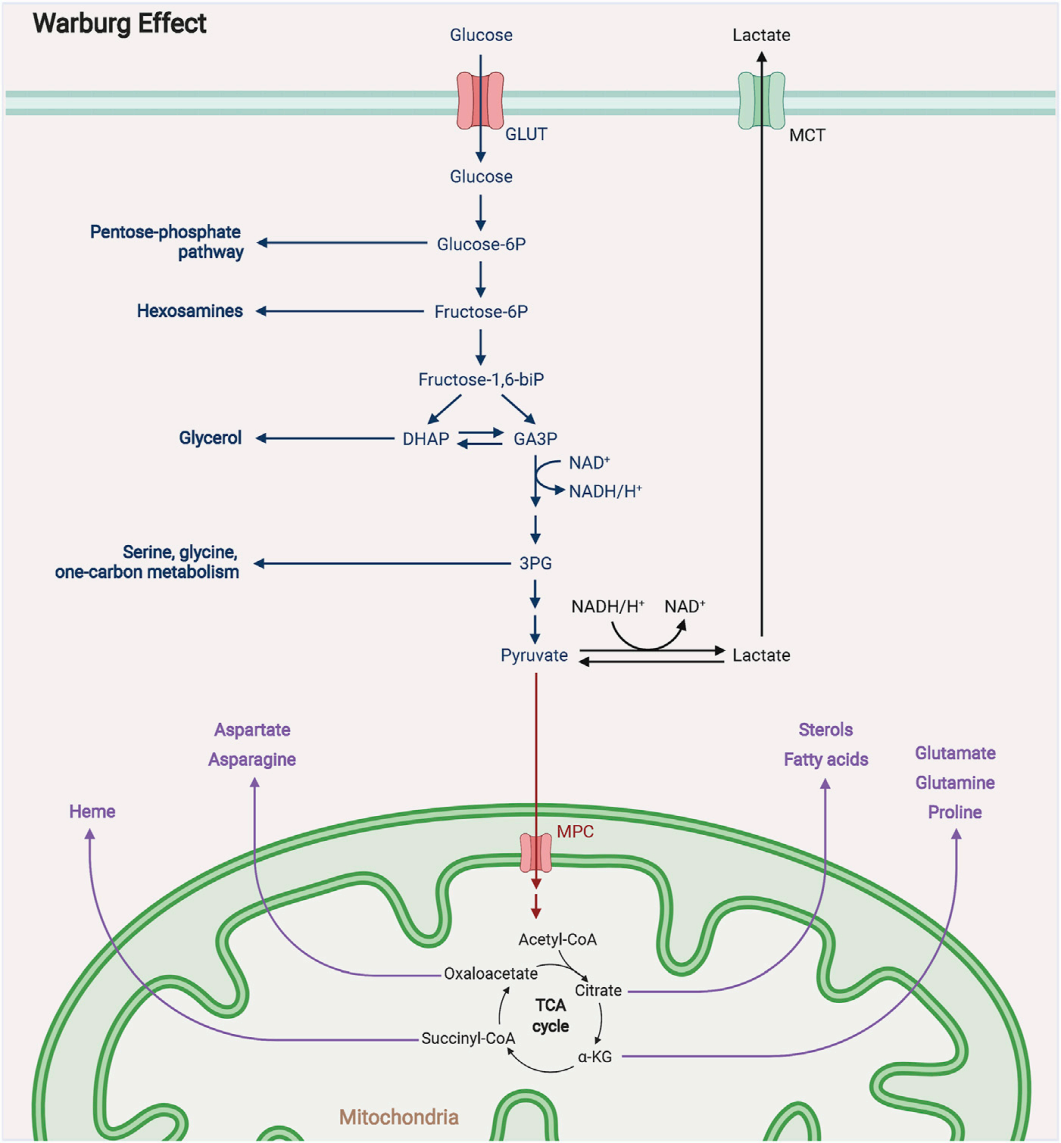
The mechanism diagram of Warburg effect. The Warburg effect states that in the presence of sufficient oxygen supply, tumor cells still prefer glycolysis for energy to the more efficient oxidative phosphorylation, a phenomenon known as the Warburg effect.

Warburg effect is mainly a compensatory activity of tumor to adapt to the external environment (Lu et al., 2015; Cassim et al., 2020) (Figure 1) Efficient aerobic glycolysis facilitates tumor cell proliferation allowing tumor cells to produce abundant ATP from extracellular nutrients. Although the total energy produced per glucose during the Warburg effect is less than that by oxidative phosphorylation, ATP production by aerobic glycolysis can exceed that of oxidative phosphorylation with glucose available (Linehan and Rouault 2013; Hitosugi and Chen 2014). On the other hand, the Warburg effect provides tumor cells with intermediates for biosynthetic pathways, including ribose for nucleotide synthesis, glycerol, citrate, and nonessential amino acids for lipid synthesis (Ward and Thompson 2012; Upadhyay et al., 2013). Glucose can also produce nicotinamide adenine dinucleotide phosphate *via* the pentose phosphate pathway. Therefore, the Warburg effect is vital for facilitating tumor cell bioenergetics and biosynthesis.

### 2.2 Factors Affecting Aerobic Glycolysis

#### 2.2.1 GLUTs

Compared with healthy cells, tumor cells exhibit an efficient aerobic glycolysis rate, which requires increased glucose flux to improve the efficiency of glucose uptake (Yang et al., 2020). Therefore, the expression and activity of Glucose Transporters (GLUTs) and glycolytic rate-limiting enzymes, such as HK, PFK and PK were significantly upregulated in tumor cells to facilitate the inevitably increased glucose consumption (Foltynie 2019; Bommer et al., 2020; Faustman 2020). Oncogenes regulate GLUT1 to intervene the glucose intake and tumor cell metabolism. The c-myc induces GLUT1 overexpression leading to increased glucose uptake (Leen et al., 2013; Huang L et al., 2021; Su et al., 2021). P53 can inhibit GLUT1 expression in cells, resulting in decreased glucose uptake and thus inhibiting tumor development (Feng et al., 2018). GLUT3 is expressed in most cancer cells but rarely in normal cells, facilitating glucose consumption (Cazzato et al., 2021; Libby et al., 2021). Targeting GLUT can inhibit the degree of aerobic glycolysis, affecting tumorigenesis (Fu et al., 2021; Kim E et al., 2021).

#### 2.2.2 HK Isoforms

Glycolysis is a complex process that starts with glucose catalyzation by various non-rate limiting and rate-limiting enzymes to form lactate (Ganapathy-Kanniappan 2018; Fan et al., 2019). The classical glycolysis involves three rate-limiting enzymes, HK, PFK, and PK, mediating different processes and playing essential roles in glucose metabolism (Shakespear et al., 2018; Yellen 2018), HK has four isoforms, HKI, HKII, HKIII, and HKIV, catalyzing glucose to glucose-6-phosphate (G6P) (Zuo et al., 2021). HKI and HKII present high affinity for mitochondria, and HK1 expression is present in most mammalian tissues (Zhong and Zhou 2017; Garcia et al., 2019). HKII is abundantly present in fat, heart, and skeletal muscle (Mathupala et al., 2009; Tan and Miyamoto 2015). with a higher glycolytic rate than HKI(Tan and Miyamoto 2015). HKIV, also known as glucokinase, is present in hepatocytes with the lowest affinity for glucose and no inhibition by G6P (Xu and Herschman 2019; Kasprzak 2021). HKII is essential for tumor metabolism. Increased expression of HKII promotes proliferation and is associated with poor prognosis in tumor patients (Roberts and Miyamoto 2015; Tan and Miyamoto 2015).

#### 2.2.3 PFK and PK

Fructose 2, 6-bisphosphate (F26BP) can diminish the inhibition of ATP and increase glucose uptake by interacting with PFK1(Kalezic et al., 2021; Zuo et al., 2021). The substrate can abnormally inhibit PFK, and ATP has a dual effect on PFK (PK is an evolutionarily conserved metabolic enzyme that catalyzes pyruvate production from phosphoenolpyruvate) (Shen et al., 2020; Zhao et al., 2020). Almost all mammalian genomes, including humans, encode two PK genes, PKLR and PKM, which express four PK isoforms (L, R, M1, and M2) (Jyoti et al., 2020; Yang et al., 2021). PKL and PKR are encoded by the PKLR gene and are expressed in hepatocytes and erythrocytes, respectively (Park et al., 2020; Storkus et al., 2021). The PKM gene encodes PKM1 and PKM2 through selective splicing (Chen k et al., 2021; Itoyama et al., 2021). PKM1 is expressed in normal differentiated tissues (Zhong et al., 2021), while PKM2 is expressed in highly proliferative cells such as embryonic cells, stem cells and tumor cells (Wang et al., 2021). Physiologically, PKM1 exists as a tetramer, while PKM2 can exist as a tetramer or a dimer (Hu et al., 2020; Rai et al., 2020). Fructose 1,6-2 phosphate is a transactivator of PKM2 but has little effect on PKM1 (Xu et al., 2019; Angiari et al., 2020).

## 3 Tumor Aerobic Glycolytic Signaling Pathway

C-myc can regulate the transcriptional process of various glycolytic genes (Gu et al., 2017). C-myc can bind to the regulatory region of hexokinase 2 (HK2) and thus play an essential role in tumor aerobic glycolysis (Huang WL et al., 2021; Su et al., 2021). PK catalyzes the final step of glycolysis, PKM2, which is only found in self-renewable groups such as stem cells and tumors (Li et al., 2017; van Niekerk and Engelbrecht 2018). C-myc can directly activate the PKM2 promoter region and upregulate PKM2 expression, thus promoting tumor aerobic glycolysis (Li et al., 2017; Yin et al., 2019). In addition, c-myc can induce PKM2 splicing by indirectly regulating hnRNP protein, thus promoting aerobic glycolysis (Gu et al., 2017). Glucose-6-phosphate dehydrogenase is a key enzyme in the glucose metabolism pathway. C-myc binds to the promoter region of glucose-6-phosphate dehydrogenase to promote its expression and thus the pentose phosphate pathway (Tang et al., 2021).

Ras-mediated metabolic reprogramming provides vital functions in tumorigenesis (Lin et al., 2021). The Ras signaling pathway can promote aerobic glycolysis and provide lactate and α-ketoglutarate through various enzymes (Campbell and Philips 2021; Chen B et al., 2021). Ras can promote glucose uptake by upregulating the expression of GLUT1 on the cell membrane surface, which in turn increases aerobic glycolysis efficiency (Healy et al., 2021). In addition, PI3K-Akt-mTOR signaling is also a significant regulator of glucose uptake, promoting GLUT1 expression and protein translocation from the inner membrane to the cell surface (Krencz et al., 2021; Sanaei et al., 2021). P53 is the most critical oncogene, affecting the cell cycle by encoding transcription factors (Liu et al., 2019; Alvarado-Ortiz et al., 2020). P53 can inhibit aerobic glycolysis by regulating TP53-mediated glycolysis and apoptosis-inducing factor expression (Strycharz et al., 2017; Itahana and Itahana 2018; Smiles and Camera 2018), regulating mitochondrial respiratory function, pentose phosphate pathway, and glycolysis-related enzymes (Kruiswijk et al., 2015; Werner et al., 2016). PTEN proteins exert their tumor-suppressive effects through three predominant signaling pathways, PI3K/AKT, local adherens spot kinase and mitogen-activated protein kinase (Mendes et al., 2016). PTEN inhibits tumorigenesis by activating PI3K/AKT pathway (Ortega-Molina and Serrano 2013). Phosphoglycerate kinase 1 (PGK1) can function as a glycolytic enzyme or phosphorylated as a protein kinase (He et al., 2019; Zhang et al., 2019). PTEN directly interacts with PGK1 to control aerobic glycolysis in tumors, and PTEN encodes a protein with phosphatase activity that inhibits phosphorylated PGK1, which ultimately inhibits aerobic glycolysis and tumor cell proliferation (Nie et al., 2020; Chu et al., 2021).

## 4 The Regulatory Mechanism of ncRNAs in the Glycolysis of Ovarian Cancer

**FIGURE 2:**
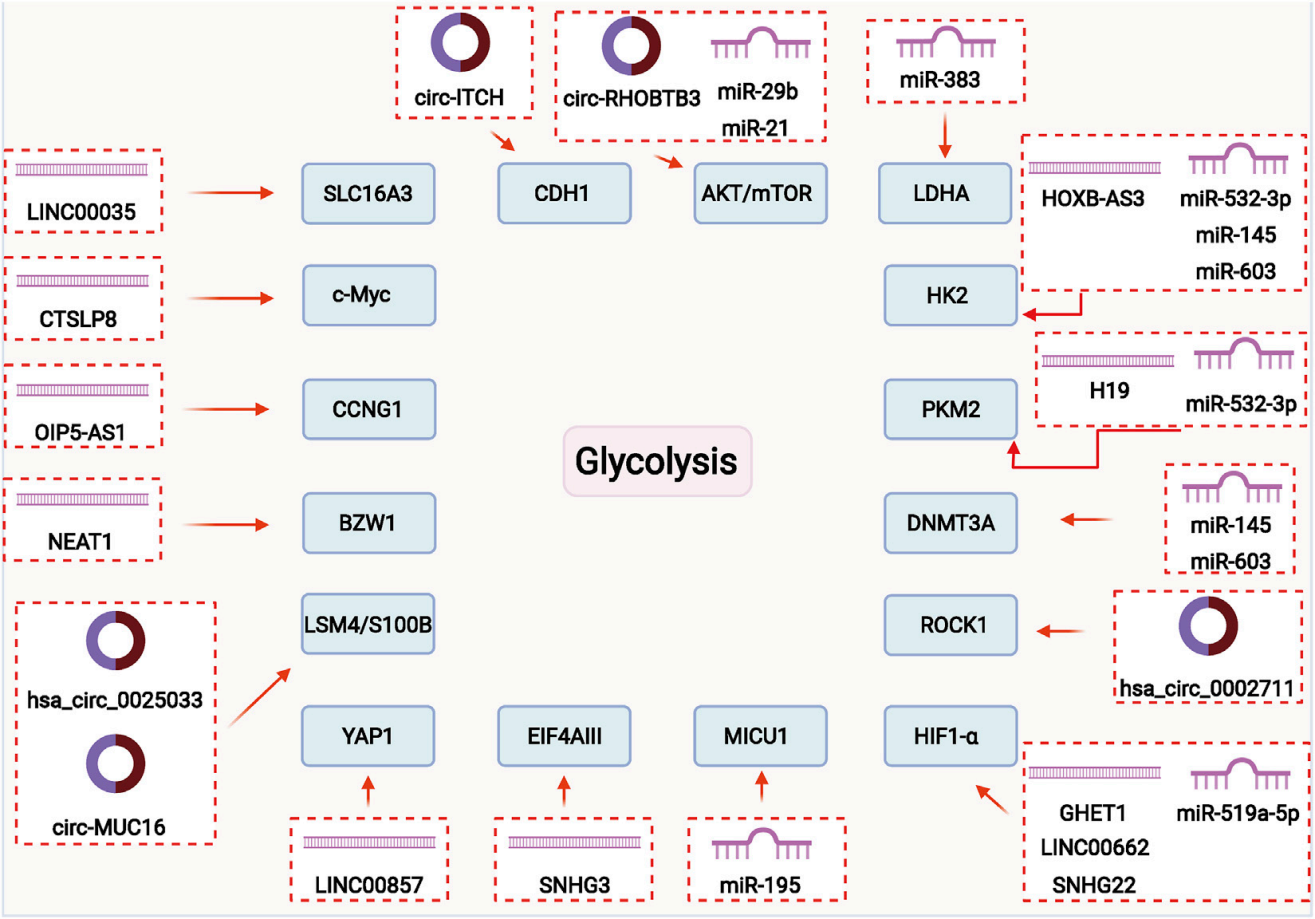
ncRNAs may play a vital role in regulating glycolysis of ovarian cancer through different signal pathways and mechanisms.

The ncRNAs can regulate the expression of criticalgenes or enzymes of glycolytic pathway through different cellular signaling pathways, which promote the malignant development by regulating glucose metabolism in OC. Here, we summarize the mechanisms of miRNAs, lncRNAs and circRNAs in the regulation of glycolysis in OC (Figure 2).

### 4.1 MicorRNAs in the Glycolysis of Ovarian Cancer

**FIGURE 3:**
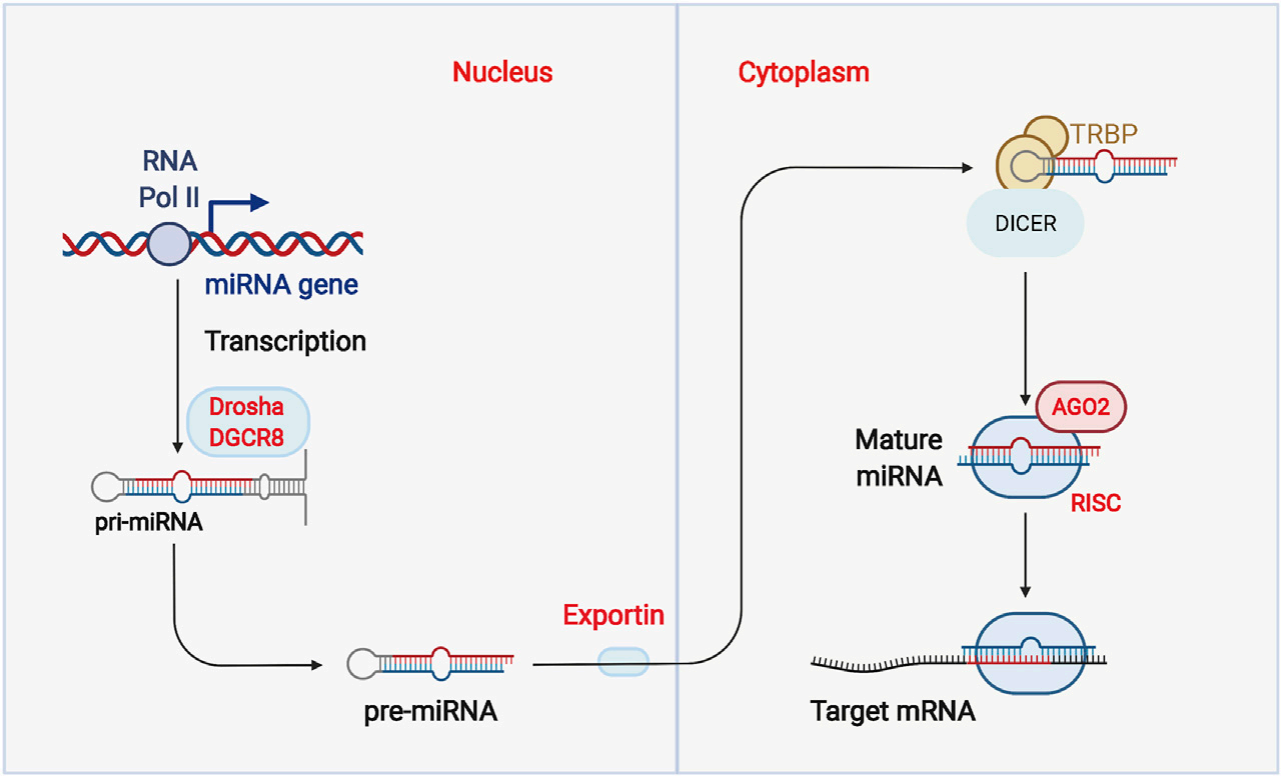
Biogenesis of micro RNAs (miRNAs). RNA polymerase II regulates the transcription of miRNAs. As pri-miRNAs are transcribed, pri-miRNAs are processed by several sequential cleavages to produce mature miRNAs. Finally, mature miRNAs are integrated into Argonaute to form the miRNA-induced silencing complex (RISC).

The miRNAs are a group of 18–24 nucleotide noncoding RNAs that bind to the 3-terminal noncoding region of the target mRNA, altering gene expression (Sakshi et al., 2021; Yang et al., 2021) (Figure 3). The aberrant expression of miRNA in tumor cells revealed that miRNAs play an essential role in tumor development by regulating the expression and function of their associated target genes and participating in a variety of physiological and pathological processes (Barrera-Rojas et al., 2021; Pidikova and Herichova 2021; Roy et al., 2021). Abundant miRNAs have been proved to regulate tumor metabolism and function as an essential role in the process of glycolysis in OC (Table 1).

**TABLE 1:** miRNAs involved in glycolysis in ovarian cancer.

MiRNAs	Role	Expression	Target	Mechanism	Type of model	References
miR-29b	Tumor suppressor	Down	AKT2/AKT3	Inhibit HK2/PKM2 expression and Warburg effect	SKOV3, A2780	Teng et al. (2015)
miR-383	Tumor suppressor	Down	LDHA	Inhibit LDHA expression	Human samples	Han et al. (2017)
miR-21	Oncogene	Up	/	Promote AKT phosphorylation and glycolysis enzymes expression	SKOV3, TOV21G	Guo et al. (2017)
miR-532–3p	Oncogene	Up	HK2 and PKM2	Inhibit HK2 and PKM2 expression	SKOV3	Zhou et al. (2018)
miR-145	Tumor suppressor	Down	HK2 and DNMT3A	DNMT3A-miR-145-HK2 regulatory axis	Human samples	Zhang et al. (2018)
miR-603	Tumor suppressor	Down	HK2 and DNMT3A	DNMT3A-miR-603-HK2 regulatory axis	/	Lu et al. (2019)
miR-1180	Oncogene	Up	/	Activate the Wnt signaling pathway	SKOV3, COC1	Gu et al. (2019)
miR-519a-5p	Tumor suppressor	Down	HIF1-α	Inhibit HK2/PKM2 expression and Warburg effect	SKOV3	Lu et al. (2020)
miR-195	Tumor suppressor	Down	MICU1	/	OVCAR4, A2780-CP20	Rao et al. (2020)

Studies have shown that miRNAs control the expression of several key enzymes of glycolysis to regulate the glycolytic process. As the critical rate-limiting enzymes of glycolysis, HK2 catalyzes the first irreversible step of glycolysis, which increases at significantly elevated levels in a variety of tumor cells. HK2 can significantly inhibit the function of mitochondria from regulating tumor growth, survival, and metastasis (Huang L et al., 2021; Yu et al., 2021). PKM2 becomes an essential component of tumorigenesis by providing a metabolic advantage that tumor cells can utilize the upstream lipids of glycolytic intermediates as precursors for lipid, amino acid, and nucleic acid synthesis (Xia et al., 2021; Yuan et al., 2021). Zhou et al., found that 20(S)-Rg3 significantly attenuated DNA methyltransferase 3 alpha (DNMT3A)-mediated methylation and promoted the inhibition of HK2 and PKM2 by miR-532–3p, thereby antagonizing the Warburg effect in OC cells (Zhou et al., 2018). Zhang et al., found that miR-145 could target DNMT3A to reduce methylation of the pre-miR-145 promoter region. The feedback loop between these two miRNA was a characteristic feature of the Warburg effect, promising a potential therapeutic target for OC(Mirzaei et al., 2016; Zhang et al., 2018). Lu et al., reported a similar regulatory machanism between miR-603 and DNMT3A, and the DNMT3A-miR-603-HK2 regulatory axis may be the critical molecular mechanism in the glycolytic pathway of OC(Lu et al., 2019; Pourhanifeh et al., 2020).

Lactate dehydrogenase A (LDHA) is an important metabolic enzyme belonging to the 2-hydroxy acid oxidoreductase family that plays a crucialrole in intracellular anaerobic sugar metabolism (Guan H et al., 2021; Huo et al., 2021). Hypoxic conditions induced the overexpression of LDHA, which shifts the metabolic pathway of ATP synthesis from oxidative phosphorylation to aerobic glycolysis. Therefore, the inhibition of LDHA is considered a promising strategy for tumor therapy (Jiang et al., 2021; Martinez-Ordonez et al., 2021). Han et al., demonstrated that miR-383 regulates LDHA expression in OC cells, impeding glycolysis, cell proliferation and invasion (Han et al., 2017). Tumor glycolytic activity is enhanced to adapt to ischemic and hypoxic environment by inducing an energy metabolic switch as the metabolic basis of its hypoxia tolerance (Wang et al., 2021). This process activateshypoxia-inducible factor-1 (HIF-1), a widely present dominant oxygen regulator in mammals, triggers various biological events, including glycolytic activation and tumorigenesis (Favier et al., 2015; Moldogazieva et al., 2020). Lu et al., reported that 20(S)-Rg3 upregulates miR-519a-5p expression by reducing DNMT3A-mediated DNA methylation of miR-519a-5p, thereby inhibiting HIF-1α and promoting the Warburg effect, leading to malignant progression of OC(Lu et al., 2020).

Aberrant activation and inactivation of oncogenes regulate abnormal energy metabolism to adapt to tumor growth demands (Yeung et al., 2008; Meijer et al., 2012). Teng et al., demonstrated that inhibition of miR-29b promotes the expression of AKT2/3, pakt2/3, HK2, and PKM2 and regulates pyruvate and NAD+/NADH levels (Teng et al., 2015). The miR-29b regulates the Warburg effect in OC by modulating AKT2/AKT3, which is a potential therapeutic target for OC. Moreover, miR-21 could promote AKT phosphorylation and glycolysis enzymes expression in OC(Guo et al., 2017). The miR-1180 could activate the Wnt signaling pathway and regulate the glycolysis progression of OC(Gu et al., 2019). Rao et al., demonstrated that miR-195 significantly inhibited tumor growth, increased tumor proliferation time, and improved overall survival by targeting MICU1 to inhibite glycolysis and chemoresistance (Rao et al., 2020).

### 4.2 LncRNAs in the Glycolysis of Ovarian Cancer

**FIGURE 4:**
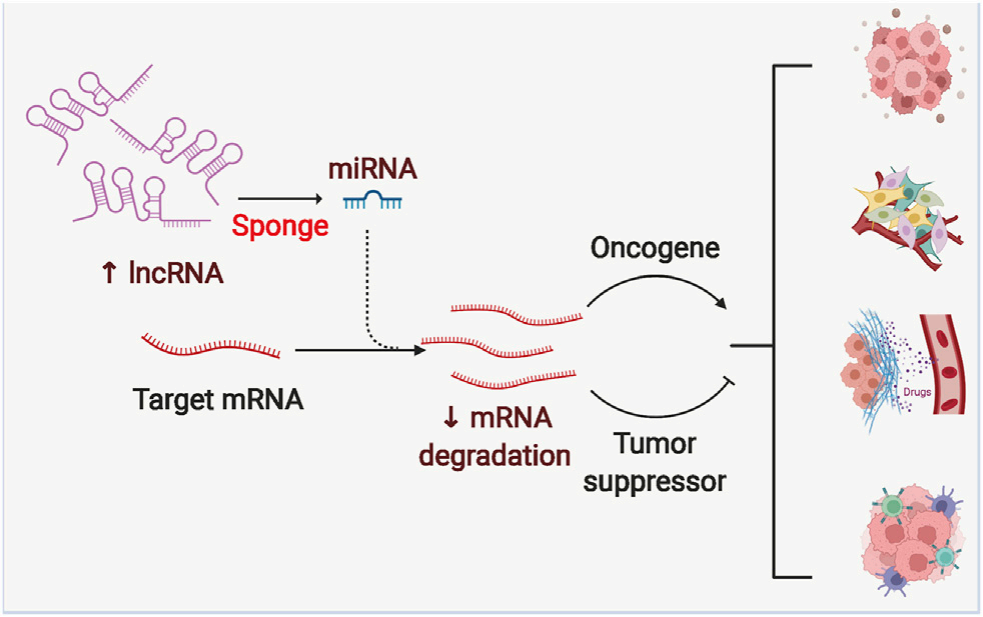
The competing endogenous RNA mechanism of Long noncoding RNAs (lncRNAs). LncRNAs can inhibit the degradation of downstream mRNAs by binding different miRNAs, which in turn regulates the expression of pro- or oncogenes, ultimately leading to malignant progression of tumors.

LncRNAs are a category of noncoding RNAs with over 200 nucleotides in length, tissue specificity and low species conservation (Jalaiei et al., 2021; Zhao et al., 2021). LncRNAs bind to proteins through their unique secondary structure to form RNA-protein complexes (Dashti et al., 2021; Janaththani et al., 2021; Mardani et al., 2021) and interact with multiple RNAs to form complex gene expression regulatory networks (Sun and Feinberg 2021; Wu et al., 2021). LncRNAs also target miRNAs through their 3′UTR region to regulate the effective concentration and activity, which affects the repressive effect on the target mRNAs(Sun and Feinberg 2021; Wu et al., 2021). (Figure 4). Above all, lncRNAs are the critical regulators in the process of glycolysis in OC (Table 2).

**TABLE 2:** lncRNAs involved in glycolysis in ovarian cancer.

LncRNAs	Role	Expression	Target	Mechanism	Type of model	References
LINC00092	Oncogene	Up	/	Bind to PFKFB2	Human samples, SKOV3	Zhao et al. (2017)
SNHG3	Oncogene	Up	miR-186–5p	Promote EIF4AIII expression	Human samples, SKOV3, TOV-21G, OVCAR3	Li et al. (2018)
H19	Oncogene	Up	miR-324–5p	Promote PKM2 expression	SKOV3	Zheng et al. (2018)
GHET1	Oncogene	Up	/	Interact with VHL and up-regulate HIF1-α	HOSEpiC, SKOV3, TOV-21G, 3AO, A2780	Liu and Li (2019)
LINC00504	Oncogene	Up	miR-1244	/	HOSEpiC, SKOV3, CAOV3, OVCAR3, HO-8910	Liu et al. (2020)
LINC00662	Oncogene	Up	miR-375	Promote HIF1-α expression	IOSE-29, SKOV3	Tao et al. (2020)
LINC00857	Oncogene	Up	miR-486–5p	Promote YAP1 expression	SKOV3, CAOV3, A2780, IOSE-29	Lin et al. (2020)
NEAT1	Oncogene	Up	miR-4500	Promote BZW1 expression	CAOV3, ES-2, iose80	Xu et al. (2020)
HOXB-AS3	Oncogene	Up	miR-378a-3p	Promote LDHA and ECAR expression	SKOV3, A2780	Xu et al. (2021)
OIP5-AS1	Oncogene	Up	miR-128–3p	Promote CCNG1 expression	IOSE-80, OVCAR-3, SKOV-3	Liu et al. (2021)
CTSLP8	Oncogene	Up	/	Promote c-Myc expression by binding to PKM2	SKOV-3, SKOV3-DDPee	Li X et al. (2021)
SNHG22	Oncogene	Up	/	SP1 and HIF1-α can promote SNHG22 expression	ES-2, HO8910, OVCAR-3, A2780	Guan N et al. (2021)
LINC00035	Oncogene	Up	/	Promote SLC16A3 expression by binding to CEBPB	IOES80, CAOV-3, A2780, SKOV3, CoC1	Yang et al. (2021)

Small nucleolar RNA host gene 3 (SNHG3) promotes glycolysis and oxidative phosphorylation to induce OC drug resistance by binding to miR-186–5p and upregulating EIF4AIII expression (Li et al., 2018). H19 promotes glycolysis and malignant progression of OC by binding miR-324–5p to promote PKM2 expression (Zheng et al., 2018). LINC00857 acts as a pro-oncogene by binding miR-486–5p to promote Yes1 associated transcriptional regulator (YAP1) expression, promoting OC cell proliferation, migration, invasion, and glycolytic progression (Lin et al., 2020). Nuclear paraspeckle assembly transcript 1 (NEAT1) can play an essential role in OC malignant growth, metastasis and glycolysis by binding to miR-4500 and thus promoting basic leucine zipper and W2 domains 1 (BZW1) expression (Xu et al., 2020). HOXB-AS3 regulates both LDHA and ECAR expression by binding to miR-378a-3p in the glycolytic process of OC(Xu et al., 2021). OIP5-AS1 binds miR-128–3p to promote the expression of CCNG1, which leads to the malignant progression of OCthrough the glycolytic process (Liu et al., 2021). Moreover, LINC00504 is involved in the glycolytic process of OC by binding miR-1244. However, the specific downstream genes need more elaboration (Liu et al., 2020).

HIF is a nuclear transcription factor that facilitates cells to adapt to the hypoxic environment (Knutson et al., 2021; Cowman and Koh 2022). Liu et al., found that upregulation of gastric carcinoma proliferation enhancing transcript 1 (GHET1) positively correlated with tumor size, metastasis, proliferation, and colony formation in OC patients (Liu and Li 2019). Further studies confirmed that GHET1 interacted with von Hippel-Lindau (VHL) to prevent VHL-mediated hypoxia-inducible factor-1α (HIF-1α) degradation and increased HIF1α protein levels in OC cells. The up-regulated HIF-1α promoted glucose uptake and lactate production in OC cells. Tao et al., reported that LINC00662 was highly expressed in OC cells and was strongly associated with overall survival of OC patients (Tao et al., 2020). Mechanistic studies confirmed that LINC00662 act as a competitive RNA to regulate HIF-1α expression by directly binding to miR-375, which in turn regulates the proliferation and glycolysis of OC cells. Guan et al., found that SP1 and HIF1-α can promote SNHG22 expression and promote the glycolytic process and malignant progression of OC(Guan H et al., 2021).

In addition, there are lncRNAs that can directly regulate the expression of genes involved in the glycolytic process of OC. LINC00092 binds 6-phosphofructo-2-kinase/fructose-2,6-biphosphatase 2 (PFKFB2) and thus promotes malignant metastasis of OC by altering glycolysis and maintaining the local support function of cancer-associated fibroblasts (CAF) (Zhao et al., 2017; Hashemipour et al., 2021). Li et al., revealed that CTSLP8 expression increases in chemoresistant tumor tissues, which promotes c-Myc expression and thus upregulates glycolysis by facilitating the binding of PKM2 to the c-Myc promoter region (Li Q et al., 2021). Yang et al., demonstrated that LINC00035 promotes malignant progression of OC by regulating glycolysis and apoptosis through CEBPB-mediated SLC16A3 transcription (Yang et al., 2021).

### 4.3 circRNAs in the Glycolysis of Ovarian Cancer

**FIGURE 5:**
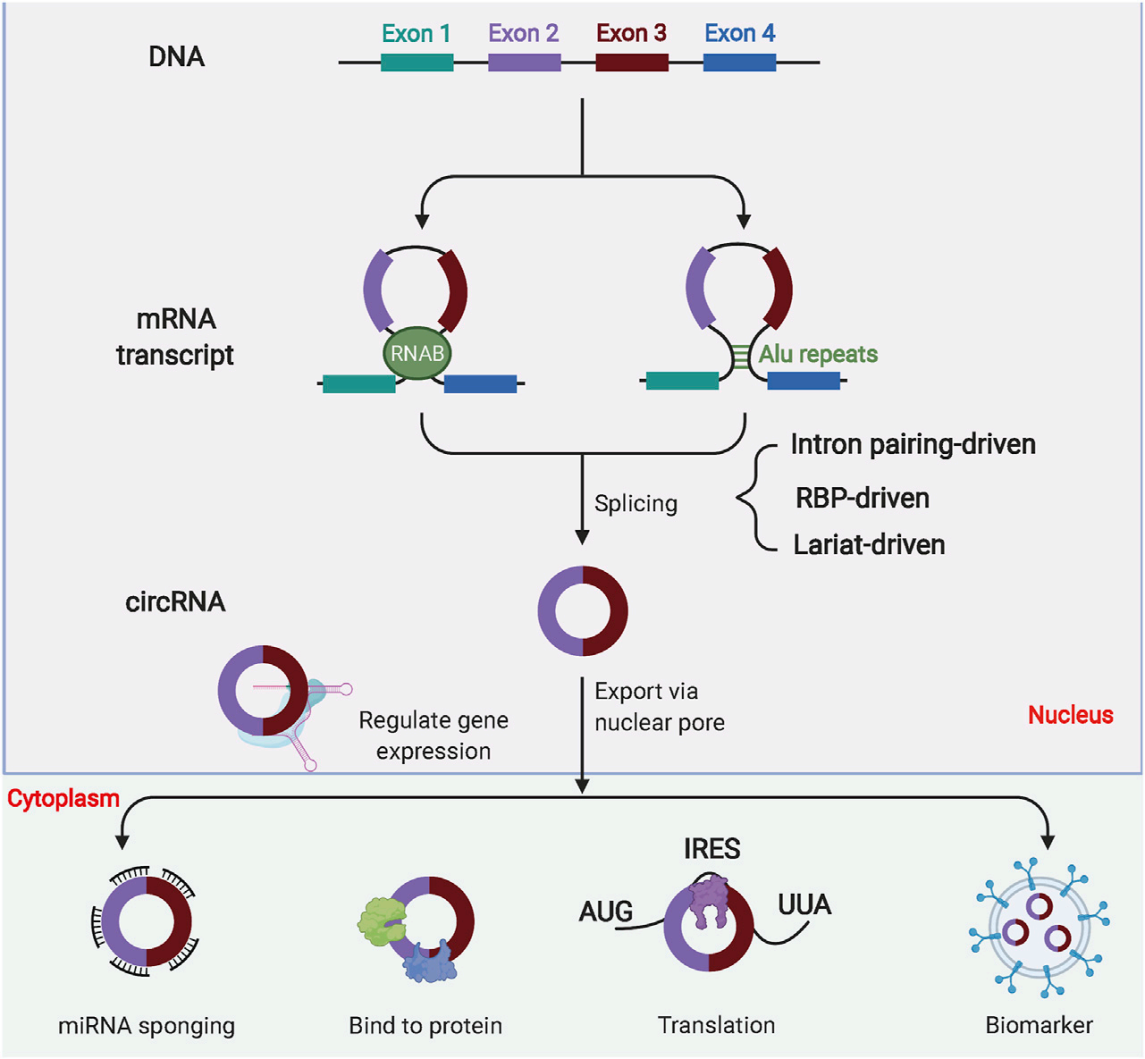
Biogenesis of circular RNAs (circRNAs). Most circRNAs are derived from pre-mRNA. Due to their composition, circRNAs are classified into several types, including exonic circRNAs, exon-intron circRNAs and intronic circRNAs. CircRNAs can perform biological functions by binding miRNAs, binding proteins or translating into polypeptides. In addition, circRNAs are also enriched in exosomes and are good markers for disease diagnosis.

Most circRNAs are expressed from known protein-coding genes and consist of exons forming a covalently closed loop structure by aberrant reverse splicing (Figure 5). CircRNA formation mechanisms included intron pairing-driven circularization, RNA-binding protein (RBP)-driven circularization, and lasso-driven circularization. The circRNAs play critical biological functions in eukaryotic organisms, which compete for miRNAs. By base-complementary pairing with the target mRNA 3-UTR, miRNAs can block the translation and stability of target RNA-binding Proteins (RBPs) can interact with circRNAs and regulate circRNA splicing, replication, folding, stabilization and localization (Huang and Zhu 2021; Zeng et al., 2021). In summary, the circRNAs act as miRNA sponges and interact with RBPs to perform transcriptional functions in organisms. The open reading frames in circRNAs enrich exosomes and can be translated into polypeptides for early diagnosis and prognosis (Kim H et al., 2021; Sinha et al., 2021; Wang et al., 2021). The circRNAs are critical in regulating the process of glycolysis in OC (Table 3).

**TABLE 3:** circRNAs involved in glycolysis in ovarian cancer.

CircRNAs	Role	Expression	Target	Mechanism	Type of model	References
Circ-ITCH	Tumor suppressor	Down	miR-106a	Promote CDH1 expression	A2780, OVCAR3, ISOE80	Lin et al. (2020)
RHOBTB3	Tumor suppressor	Down	/	Inactivate PI3K/AKT signaling pathway,	IOSE-80, OVCAR-3, SKOV-3	Yalan et al. (2020)
				Inhibit GLUT1, HK2 and LDHA expression		
Hsa_circ_0025033	Oncogene	Up	miR-184	Promote LSM4 expression	A2780, OVCAR3, ISOE80	Hou and Zhang (2021)
Hsa_circ_0002711	Oncogene	Up	miR-1244	Promote ROCK1 expression	OVCAR-3	Xie W et al. (2021)
Circ-MUC16	Oncogene	Up	miR-1182	Promote S100B expression	A2780, SKOV-3, ISOE80	Yang GJ et al. (2021)

Circ-ITCH was downregulated in OC and positively correlated with 5-years overall survival in OC patients (Lin et al., 2020) while the overexpression significantly inhibited proliferation, invasion, glycolysis and promoted apoptosis in OC cells. Sun et al., demonstrated the downregulation of circ-RHOBTB3 in OC tissues and cells, and overexpression significantly inhibited cell proliferation, metastasis, and glycolysis (Yalan et al., 2020). Circ-RHOBTB3 inhibited OC progression by inactivating the PI3K/AKT signaling pathway. The expression of hsa_circ_0025033 was found to be upregulated in OC, and downregulation of hsa_circ_0025033 significantly inhibited OC cell colony formation, migration/invasion and glycolytic metabolism (Hou and Zhang 2021). Hsa_circ_0025033 promotes LSM4 expression by binding miR-184. Xie et al., demonstrated that the hsa_circ_0002711/miR-1244/ROCK1 regulatory axis promotes malignant progression of OC *in vivo* by regulating Warburg effect and tumor growth (Xie W et al., 2021). Hsa_circ_MUC16 promotes OC cell proliferation, glycolytic metabolism, migration and invasion by targeting the miR-1182/S100B regulatory axis (Yang et al., 2021).

## 5 Future Perspectivesand Conclusion

The development and progression of OC is a complex physiological process. The invasion and metastasis of OC is a complicated process, which poses difficulties for early detection, intervention, and treatment (Tymon-Rosario et al., 2021; Wang et al., 2021). The Warburg effect is one of the recognized metabolic features of tumor cells (Abi Zamer et al., 2021; Nakagawa et al., 2021). Active glycolysis remains a common feature of cancer metabolism, and metabolic reprogramming increases the expression of critical enzymes and, ultimately, lactate secretion. Lactate in the tumor microenvironment can promote malignant progression and tumor immune escape (Hashemian et al., 2020; Mirzaei and Hamblin 2020; Holloway and Marignani 2021; Nakagawa et al., 2021). Various oncogenes and signaling pathways regulate the glycolytic enzymes to affect the rate of glycolysis (Almeida et al., 2021; Chandel 2021). Although the glycolytic process has drawn attention in the control of oncogenic features, the mechanisms of critical enzymes and complex interactions with signaling are not well studied in OC, considering the high heterogeneity of tumors.

Findings have confirmed the regulatory role of ncRNAs on the Warburg effect of tumor cells and highlight their significance in tumor biology research. The expression of specific ncRNAs in tumors predicts tumors’ biological properties and their possible outcomes and prognosis. On the other hand, ncRNAs may also become target sites for tumor treatment. However, there are still relatively few discoveries lacking systematic content and reliable clinical evidence. In summary, ncRNAs play an essential role in OC aerobic glycolysis, regulating the activity and content of specific enzymes and acting as transcriptional activators to regulate the expression of metabolism-related genes. In addition, these ncRNAs interact with other critical factors related to glucose metabolism and initiate various oncogenic processes. In the future, it is vital to confirm and elucidate the role of ncRNAs in OC aerobic glycolysis and their potential as molecular biomarkers. Investigating the correlation of ncRNA and aerobic glycolysis is promising for the interaction network of ncRNAs and the feedback regulation in tumorigenesis. Elucidating the mechanism of ncRNAs in the aerobic glycolysis of OC will provide new insights into OC research and provide new strategies for clinical treatment.

## Abbreviations

BZW1, basic leucine zipper and W2 domains 1; circRNAs, circular RNAs; CAF, cancer-associated fibroblasts; DNMT3A, DNA methyltransferase 3 alpha; F26BP, Fructose 2, 6-bisphosphate; GHET1, gastric carcinoma proliferation enhancing transcript 1; HK2, hexokinase 2; HIF-1, hypoxia-inducible factor-1; lncRNAs, long non-coding RNAs; LDHA, lactate dehydrogenase A; miRNAs, microRNAs; ncRNAs, Non-coding RNAs; NEAT1, nuclear paraspeckle assembly transcript 1; OC, Ovarian cancer; PKM, pyruvate kinase M1/2; PFKFB2, 6-phosphofructo-2-kinase/fructose-2,6-biphosphatase 2; RBP, RNA-binding protein; SNHG3, small nucleolar RNA host gene 3; VHL, von Hippel-Lindau; YAP1, Yes1 associated transcriptional regulator.
